# Integrated sequence and immunology filovirus database at Los Alamos

**DOI:** 10.1093/database/baw047

**Published:** 2016-04-21

**Authors:** Karina Yusim, Hyejin Yoon, Brian Foley, Shihai Feng, Jennifer Macke, Mira Dimitrijevic, Werner Abfalterer, James Szinger, Will Fischer, Carla Kuiken, Bette Korber

**Affiliations:** Los Alamos National Laboratory, Los Alamos, NM, USA

## Abstract

The Ebola outbreak of 2013–15 infected more than 28 000 people and claimed more lives than all previous filovirus outbreaks combined. Governmental agencies, clinical teams, and the world scientific community pulled together in a multifaceted response ranging from prevention and disease control, to evaluating vaccines and therapeutics in human trials. As this epidemic is finally coming to a close, refocusing on long-term prevention strategies becomes paramount. Given the very real threat of future filovirus outbreaks, and the inherent uncertainty of the next outbreak virus and geographic location, it is prudent to consider the extent and implications of known natural diversity in advancing vaccines and therapeutic approaches. To facilitate such consideration, we have updated and enhanced the content of the filovirus portion of Los Alamos Hemorrhagic Fever Viruses Database. We have integrated and performed baseline analysis of all family *Filoviridae* sequences deposited into GenBank, with associated immune response data, and metadata, and we have added new computational tools with web-interfaces to assist users with analysis. Here, we (i) describe the main features of updated database, (ii) provide integrated views and some basic analyses summarizing evolutionary patterns as they relate to geo-temporal data captured in the database and (iii) highlight the most conserved regions in the proteome that may be useful for a T cell vaccine strategy.

**Database URL**: www.hfv.lanl.gov

## Introduction

Since their discovery in 1967 (1), viruses in the family *Filoviridae* have caused multiple lethal human disease outbreaks.

Viruses belonging to five species in the *Ebolavirus* genus, Ebola virus (EBOV), Sudan virus (SUDV), Reston virus (RESTV), Taï Forest virus (TAFV) and Bundibugyo virus (BDBV) cause Ebola virus disease (EVD); viruses in two distinct lineages in the *Marburgvirus* genus, Marburg virus (MARV) and Ravn virus (RAVV) cause Marburg virus disease (MVD) (2). The first filovirus discovered, MARV, originated from a zoonotic transmission from infected monkeys shipped from Uganda (1); it caused a lethal human MVD outbreak in 1967 in Marburg and Frankfurt, West Germany (now Germany), and a related, nearly simultaneous outbreak in Yugoslavia (now Serbia). The ebolaviruses were discovered in 1976 during an EVD outbreak due to EBOV infection in Zaire (now the Democratic Republic of the Congo). Nearly 50 documented EVD and MVD outbreaks of relatively limited sizes occurred over the next several decades, but in 2013 a child in Guinea became the index case of an Ebola disease epidemic in Western Africa that spread through multiple nations. This outbreak has spanned 3 years, and infected >28 000 people (3). The response to support afflicted regions has been global (4 –12), and historically, citizens of many nations have been touched directly by these outbreaks. Our database provides an ebolavirus and marburgvirus global map that tracks the origin of EVD and MVD outbreaks according to zoonosis, human migration, import of non-human primates and laboratory-accident infections (Supplementary Figure S1).

There are several useful web-based resources for accessing data and conducting analysis of filoviruses, especially ebolaviruses. World Health Organization (WHO) and Centers for Disease Control (CDC) include lists of EVD and MVD outbreaks, up-to-date statistics, maps and response data covering the 2013–15 EVD outbreak in Western Africa, as well as general factsheets and disease information (13–15). The Immune Epitope Database (IEDB) lists immunological responses to a wide variety of pathogens, including filoviruses (16). The University of California, Santa Cruz (UCSC) Ebola Genome Portal hosts the Ebola Genome Browser with viral sequences from previous and current EVD and MVD outbreaks, as well as related data, literature and analysis links (17). The NCBI Virus Variations Resource contains sequence and taxonomy data and a search interface (18). The Ebolavirus Virus Pathogen Resource hosts data and web-based tools for sequence and structure analysis, comparative genomics and phenotype studies (19). The Hemorrhagic Fever Viruses (HFV) Database at Los Alamos National Laboratory (www.hfv.lanl.gov) (20), extant from 2009, includes filoviruses; for the past few years, due to lack of funding, this database was not actively curated other than minimal integration of new sequences as they appeared in GenBank. In response to the 2013–15 EVD outbreak, short-term funding became available for updating and enhancement of the filovirus section of the HFV database. Our Filovirus Database complements other web-based filovirus resources: specifically, we facilitate the integration and analysis of published filovirus sequence data, immune response data and metadata, and provide ready access to information capturing the diversity of any part of the genome or proteome based on web queries. This is accomplished through a searchable relational database and web-based analysis tools, many of which are tailored specifically for application to filovirus analysis (Supplementary Table S1).

## Methods

### HFV database resources

**Figure 1.**
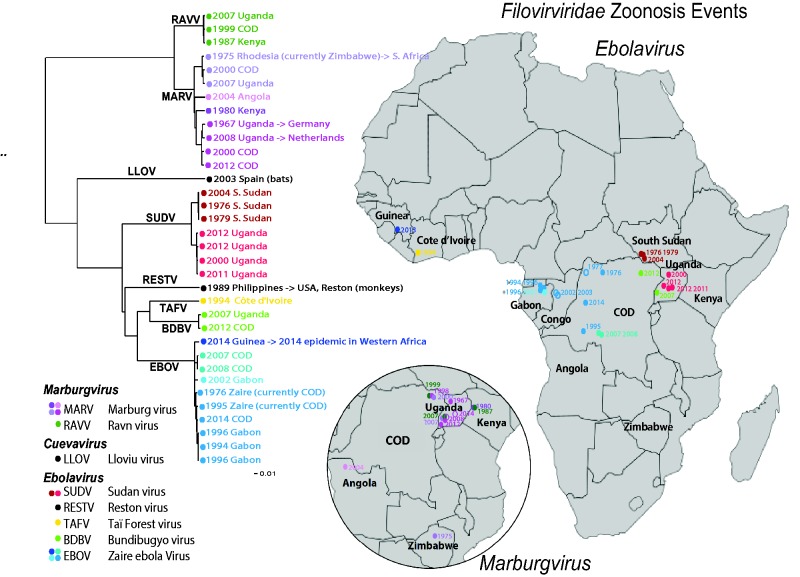
Human outbreak map and phylogeny of filoviruses. Using just the concatenated coding regions of the 34 one-per-outbreak sequences from the database (excluding the non-coding regions as they vary in length, and are very difficult to reliably align across species), we reconstructed the phylogeny of all filovirus outbreak sequences (see also Supplementary Figure S1), using the PHYML web interface provided through the HFV database (37) (http://hfvdev.lanl.gov:9100/content/sequence/PHYML/interface.html). Here, the year and the country name for each sequence are given to highlight the timing of a source of the sampled outbreak. Details concerning the sequences included in this tree and phylogenetic methods are shown in Supplementary Figure S1. If a virus was contracted by an individual while visiting a country, but discovered upon return to their own county, it is labeled with an arrow. For example a South African was infected with Marburg virus while visiting Rhodesia (now Zimbabwe) in 1975, and so the taxon representing this infection is labeled ‘ZW -> ZA 1975’ where ZW is the ISO 2 letter code for Zimbabwe, ZA is the code for South Africa, and 1975 is the year of sampling. It corresponds to the point in Zimbabwe on the Marburg inset map, labeled 1975. Of note, if the year of sampling of the sequence in the tree is later than a corresponding year in the map, it is because the disease outbreak spanned a year. For example, the 2013–15 EVD outbreak in Western Africa began with an index case infected in December 2013, so it is labeled 2013 on the map; as the first samples that were sequenced were obtained in March 2014, 2014 is indicated in the tree. The geographic source of each sequence and the time of sampling were used to associate the sequences with the disease outbreak lists (CDC links). Several of the human disease outbreaks listed by the CDC did not have a corresponding sequence, and those are noted with a year in the map, but are represented as open oval; filled ovals represent disease outbreaks with a corresponding sequence. In some cases the precise location of an outbreak is not known; for instance, the location within Uganda where the African Green Monkeys that first carried MARV to Germany is just represented by a point in Uganda; some outbreaks were only noted to be within a particular province. Viruses from different species are assigned different colors, and clades representing phylogenetically closely related sequences are indicated by similar colors.

The Filovirus Database originated as part of the larger biothreat HFVs database, which covers viruses belonging to over 80 viral species, comprising five different families: *Arena*-, *Bunya*-, *Flavi*-, *Filo*- and *Togaviridae* (20). The HFV database uses the same framework as the HIV and Hepatitis C Virus Los Alamos databases (21, 22), and many of the tools are similar, although some are tailored to a specific virus in each database. The recent annotation and enhancement effort described here covers only viruses in the family *Filoviridae* (a representative phylogeny is shown in Figure 1); however, basic sequence searches, alignments and many of the analysis tools are also applicable for the rest of the HFV Database.

### Computational framework and sequences

**Figure 2.**
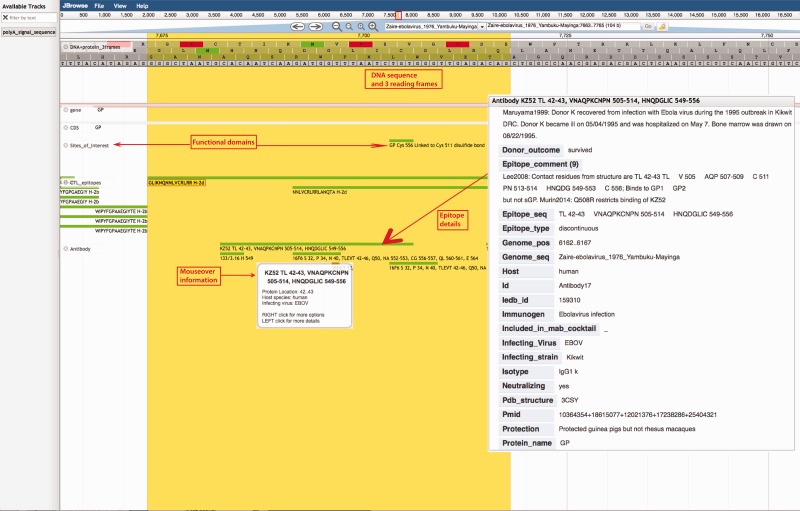
Ebola Genome Browser. http://hfv.lanl.gov/content/sequence/genome_browser/browser_ebola.html. Provides interactive viewing of the ebolavirus gene map, including functional domains and epitopes from both ebolavirus and marburgvirus. The tool is a customization of JBrowse (http://jbrowse.org/) (24, 25), built to incorporate multiple sources of information about ebolaviruses and marburgviruses. All nucleotide and protein positions shown are based on reference sequence Yambuku-Mayinga (accession NC_002549). The comments on a figure are shown with red boxes and red arrows.

The Filovirus Database uses a PostgreSQL database management system. As of this writing (January 2016), the sequence database consists of near 1700 sequences, including 1361 ebolavirus, 336 marburgvirus and 2 cuevavirus sequences, and 24 tables containing related metadata. Our database and analysis tools were built using HTML, Javascript, Mason, Perl and CGI:Perl. Sequences of filoviruses and other HFV are downloaded monthly from GenBank, and subjected to automated and manual quality control processes. The stored sequences are linked to curated descriptive information from the literature. Sequences can be accessed through the web-based interfaces, and users can tailor queries to relevant subsets of the data, to focus on specific regions of the genome or proteome. The reference sequences of each species and the annotation obtained from NCBI’s RefSeq database (23) are utilized across our site, both for the alignments and computational tools. Our Filovirus Genome Browser uses JBrowse to visualize genomic data (24, 25) (Figure 2).

### Sequence data and database searches

Upon upload, all new filovirus sequences from GenBank are put through automated quality control procedures, automatically aligned to reference sequences, and then linked to manually annotated metadata from the literature, including information regarding the hosts, geographic region and date of sampling, and published patient information such as disease outcome, patient age, gender, symptoms and dates of symptoms or clinical signs from disease onset through death or recovery. Other information relating to the virus is included, such as whether it was directly sequenced from a clinical specimen, had undergone tissue/cell culture passaging, or was adapted in the laboratory to cells or animals it would not normally infect (2, 26). The sequences can be accessed via a user-friendly interface that allows searches on > 30 such fields. The search interface also enables the exclusion of problematic sequences (e.g. synthetic, identical-to-reference sequences, sequences from patent applications or fragments under a given length). Species and viruses are named following the most recent nomenclature (26– 30). Users can design tailored sequence names with additional metadata concisely incorporated to facilitate subsequent analysis. Supplementary Figure S2 shows a sequence phylogeny with database-derived sequence names including the isolation date and the most distinguishing geographic location of the sample; an alternative display of this phylogeny, linking the information in Supplementary Figure S2 to a map of Africa, is shown in Figure 1. Annotation metadata can be also downloaded as tab-delimited tables. Search results can be sorted and selected in different ways, for example, by start position, and visualized by icons that show at a glance the length and genomic start and end positions of each sequence (Supplementary Figure S3). A graphical overview showing the distribution of the frequencies of sequences of different genera and species across the HFV genome can be created for any search set (Supplementary Figure S4). An alternative geography search interface provides a clickable map that allows users to retrieve the sequences and plot the frequencies of viruses belonging to different filovirus species on the basis of their geographical origin (Supplementary Figure S5 A. World Map, S5 B. Africa Map).

### Premade filovirus sequence alignments

Whole-genome as well as individual gene and protein sequence alignments are available for all filoviruses, as well as separate alignments for ebolavirus and marburgvirus sequences. Automated alignment using MAFFT (31) serves as a basis for these alignments, which are hand checked to determine if both the boundaries of internal gap regions and boundaries encompassing codon regions are sensible, and to resolve obvious errors. Filovirus reference sequences (2) for the viruses belonging to each of the known *Ebolavirus* and *Marburgvirus* species were used as the basis for the within species virus alignments; the species virus alignments were then aligned to each other, using the Ebola virus/H.sapiens-tc/COD/1976/Yambuku-Mayinga isolate (NC_002549) as the master reference sequence. The comprehensive alignments contain all full-length sequences available up to the last database update. The species reference alignments contain only eight filovirus reference sequences (2). The ‘outbreak’ filovirus alignments contain 34 sequences, each representing a virus that seeded a distinct human EVD or MVD outbreak (full length sequences are not available for all outbreaks) (Figure 1 and Supplementary Figure S2). For the outbreak sets, a sequence from the earliest sample in an outbreak was selected when temporal data were available; if multiple isolates were sequenced, we picked a sequence identical or closest to the consensus from the first time point, in an attempt to approximate the index case as closely as possible. To capture the known extent of the diversity of the filoviruses, RESTV and LLOV were included in the outbreak set, although these have not been isolated from human beings. Using the outbreak alignment, in Table 1 we genetic distances between virus isolates within each species, between species and between genera.

**Table 1.** Sequence differences between outbreaks within and between species

	Non-coding regions	Coding regions
Length range (bases)	4259–4444	14 451–14 607
Virus differences within species (%)*		
EBOV	3.5	1.8
SUDV	1.5	0.9
MARV	10.9	5.9
Virus differences between species (%)**		
EBOV to SUDV	64.1	35.2
EBOV to MARV	68.5	52.9

* Median differences between representative outbreak sequences compared to each other within species.

** Median differences between representative outbreak sequences in two species.

The alignments maintain the proper reading frame for all proteins in the genome, although the glycoprotein (GP) has an additional complication. The expression of the full-length membrane-bound GP trimer of Ebolavirus and Cuevavirus GPs requires the insertion of an extra adenine residue (an eighth adenine in a string of adenines) during transcriptional editing (32); otherwise a soluble glycoprotein (sGP) is produced. Addition of two adenines or subtraction of one adenine can result in the production of yet another version, ssGP. sGP is produced 70% of the time, GP 25% and ssGP 5% (33). We insert gaps in our full-length genome alignments to maintain the full length GP reading frame. For the full length GP coding regions, we add the adenine to keep the reading frame intact, as it would be in the GP mRNA. The lysine encoded by the insertion codon is also included in the GP protein alignments. Of note, marburgviruses have the extra adenine residue in the genomic RNA, and simply encode a full length GP transcript; selection for the EBOV Kikwit variant with eight adenines has also been demonstrated in cell culture (34, 35).

### Ebola genome browser immunology and genome coordinates resources

A snapshot of the genome browser resource is shown in Figure 2. Genome coordinate data are depicted for each position in the ebolavirus genome, with coding regions and functional features and domains noted. Detailed immunological data can be accessed through the genome browser. Epitope database entries include detailed information describing how each epitope was first characterized, vaccines used to elicit immune responses, continuous and conformational antibody epitope binding details, notes on structure, cross-reactivity, neutralization, therapeutic and protection comments. Pubmed references, IEDB entries and database tools aligning the epitope are all linked via the browser. Epitopes can also be visualized via epitope maps, with each epitope presented in its proteomic/genomic location relative to the alignment of eight filovirus reference sequences (2), (Supplementary Figure S6).

## Results

Here, we present some simple informative analyses to illustrate the integration of HFV database information and alignments with the database web tools. In addition to the Ebola genome browser, the database has about 30 computational tools to assist with sequence manipulation, format and display, statistical and genetic signature analysis, phylogenetic analysis and geographic distributions (Supplementary Table S1). These tools were generally adapted from the Los Alamos HIV database; some are general and can be applied to any organism, but are particularly useful here, while others have been specifically adapted to filoviruses. Some examples: *HFV BLAST* finds sequences similar to the query sequence within the HFV database. HFV Sequence Locator finds the coordinates of the query sequence with respect to the appropriate reference sequence. Genome Mapper generates and displays maps of genomic features for the selected species. Ebola Quickalign aligns short input query sequences (epitopes, functional domains, primers, binding sites or any local region of interest) to the Filovirus Database premade alignments, or to the user’s alignment, and summarizes the variability observed at the location of the query sequence. Quickalign processes short continuous query sequences, or discontinuous positions of interest, e.g. to assess variability of conformational antibody epitopes. AnalyzeAlign shows sequence logos (36), calculates frequency by position and finds variants in an alignment.

### Linking phylogenetic, geographic and temporal data among outbreak sequences

We used the HFV database metadata and sequence data to compare temporal, geographic and phylogenetic relationships between filoviruses (Figure 1 and Supplementary Figure S1). While temporal geographic maps are available at the CDC website (13, 14), the new analysis presented here puts geo-temporal data in the context of the sequence evolutionary patterns. A maximum likelihood tree was built using our outbreak alignment as input for the HFV database interface for PhyML (37). The tree was based on only coding regions, which can be much more readily aligned across genera than the highly divergent variable length inter-genic regions (Table 1). When the phylogenetic tree was superimposed to the geo-temporal map of the outbreaks (Figure 1), two distinctive patterns were evident. The first pattern is that contemporary, or near-contemporary, geographical clusters and outbreaks can result from separate introductions of diverse local viruses into humans. This pattern is well documented in the literature: for example in Durba, Democratic Republic of the Congo, former Zaire, (COD), between 1998 and 2000, there was a series of nine introductions of distinct viruses into humans, with different MARV and RAVV sequences among them (38) (See the northwest corner of the COD in the insert in Figure 1). Similarly, in 2007, both MARV and RAVV viral infections were identified in miners who worked in the Kitaka cave mines in Uganda; the sequences in the human cases each very closely matched MARV and RAVV isolated from bats in the Kitaka cave, indicating two separate introductions into humans from a single local bat colony that harbored two very distinct marburgviruses (39). In a later investigation, bats that inhabit Python Cave, where a Dutch and an American tourist where infected in 2008, were also found to carry both MARV and RAVV (40). Viruses isolated from Python Cave bats and Kitaka Cave bats were genetically very similar, and two bats that had been tagged in the Kitaka cave were later identified in the Python Cave, suggesting that bats could readily move virus between sites (40). Thus, bats can harbor diverse marburgviruses that can be introduced into local human populations in the same time frame.

The second pattern evident from the phylogenetic tree involves the phylo-geographic relationships previously documented among filoviruses. Outbreak viruses from the same region may be highly similar, with regional relationships maintained over many years (41). This is consistent with the presence of relatively stable regional reservoirs that are likely to be the source of multiple human outbreaks. A clear example of this can be seen in the three EVD outbreaks due to SUDV in S. Sudan (42). The isolates are highly similar, but were sampled over a period spanning 28 years between 1976 and 2004 (red dots in Figure 1); their high similarity is clearly associated with geographical origin rather than year of sampling. The Ugandan SUDV samples, though overlapping in time, form a cluster that is phylogenetically and geographically distinct from the samples taken in S. Sudan (Figure 1). In another example, the MARV that was transferred to Europe from Uganda in 1967 was genetically most similar to a sequence obtained from a Dutch tourist infected while visiting Uganda in 2008, 41 years later (Figure 1). Although the precise location of the 1967 infection source within Uganda was not clearly documented in the literature, the virus was more similar to a substantially later Ugandan MARV sequence than to MARV samples obtained from other countries in the intervening years, again suggesting that geographic locality was a stronger predictor of genetic similarity than was closeness in time. As previously reported (43), the sequence from the first EVD outbreak due to EBOV in 1976 in Zaire, now the Democratic Republic of the Congo (COD), (44) was much more similar to a sequence sampled in COD in 2014 EVD outbreak (43) than the sequences from the 2013–15 Western African EVD outbreak. As in the case of MARVs, although there were 38 years between the original and recent EVD outbreaks in COD (Supplementary Figure S7), the 2014 EBOV COD isolate is genetically more similar to the 1976 isolate than it is to many viruses isolated during EBOV-caused EVD outbreaks in the intervening years (Figure 1 and Supplementary Figure S7).

Taken together, these observations illustrate that while in some cases diverse viruses may co-circulate in one region at one time, in other cases viruses from a particular lineage within a species can be relatively stable in local region reservoir population, seeding EVD and MVD outbreaks over decades. Both these factors complicate interpreting the use of genetic distances between filoviruses from different outbreaks to estimate rates of evolution and time to the most recent common ancestors (42), as the observed distances may be less a function of time than of the regional events that result in particular reservoir populations seeding an outbreak. The international effort that enabled rapid sampling and sequencing of the Western Africa’s 2013–15 outbreak sequences (43, 45, 46) has allowed an unparalleled view of evolution of EBOV as it moved through a human host population (3), enabling a deeper understanding of both the epidemic in Western Africa and the biology of the virus in human host populations.

### Protein regions that are conserved and under negative selection across species and genera

**Figure 3.**
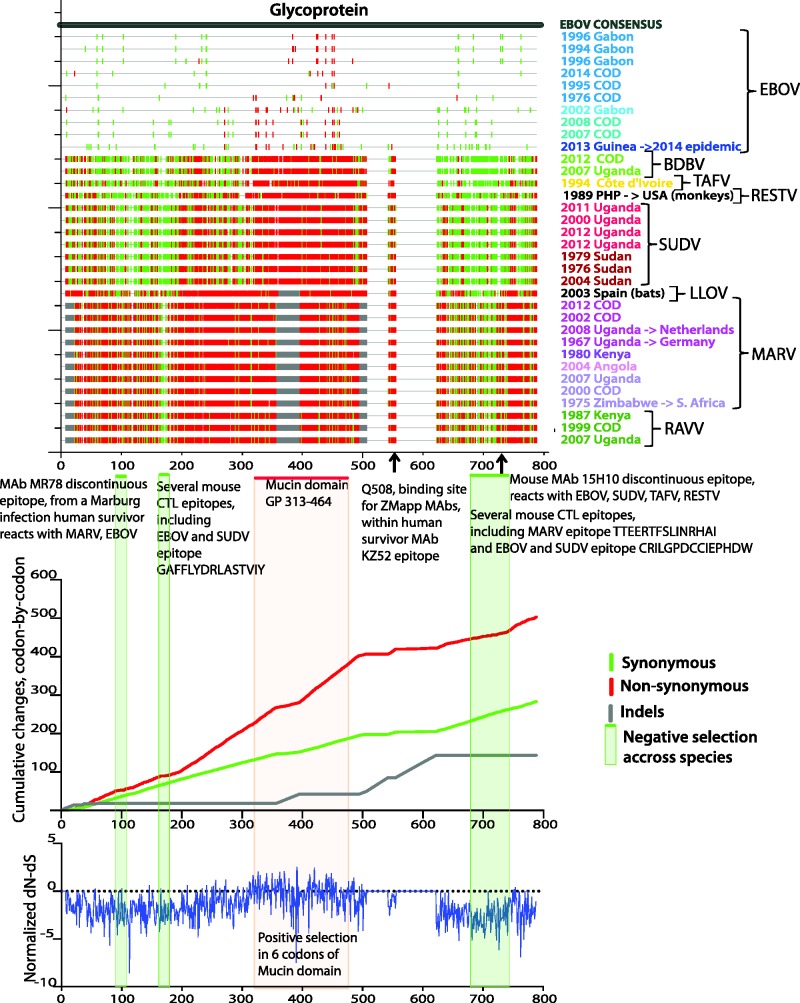
Analysis of synonymous/non-synonymous substitution rates in the GP across filoviruses. The same set of 34 one-per-outbreak sequences is used as in Figure 1 and Supplementary Figure S1. The sequences are presented in the order of the phylogenetic tree in Supplementary Figure S1, bottom to top. Sequence colors correspond to the sequence colors on Figure 1 and Supplementary Figure S1. Top panel: Created with the HFV database tool Highlighter (http://hfv.lanl.gov/content/sequence/HIGHLIGHT/highlighter_top.html). Synonymous (green) and non-synonymous (red) substitutions in each sequence with respect to EBOV consensus sequence are shown by vertical bars. Grey bars represent insertions and deletions. Codon numbering corresponds to the alignment numbering. Green horizontal bars and green-shaded areas represent regions that with negative selection (prevalent synonymous substitutions) across filoviruses. Red horizontal bar and red-shaded area show EBOV mucin-like domain. Epitopes overlapping regions of negative selection are shown under top panel’s figure. Middle panel: Cumulative synonymous (green), non-synonymous (red) and indels (gray) changes codon-by-codon, obtained using the HFV database tool SNAP (http://hfv.lanl.gov/content/sequence/SNAP/SNAP.html) are shown. Rapidly rising regions (i.e. mucin-like domain) in non-synonymous plot compared with synonymous plot correspond to high accumulation of non-synonymous changes. Slowly changing regions on non-synonymous plot compared with corresponding regions on synonymous plot indicate negative selection. Bottom panel: Normalized dN-dS difference, calculated with the external DataMonkey SLAC tool (http://www.datamonkey.org/), (48– 50). The general reversible substitution model was selected by Datamonkey out of 199 models. The analysis was run using single-likelihood ancestor counting (SLAC), fixed effects likelihood (FEL), random effects likelihood (REL) methods, and the integrated analysis, as recommended by Kosakovsky Pond *et al.* (50). The three methods combined found 6 sites of positive selection, supported by two or all three methods, in or very close to the mucin-like domain region, in codon positions 309, 310, 318, 332, 403, 430, relative to EBOV Mayinga reference isolate.

**Figure 4.**
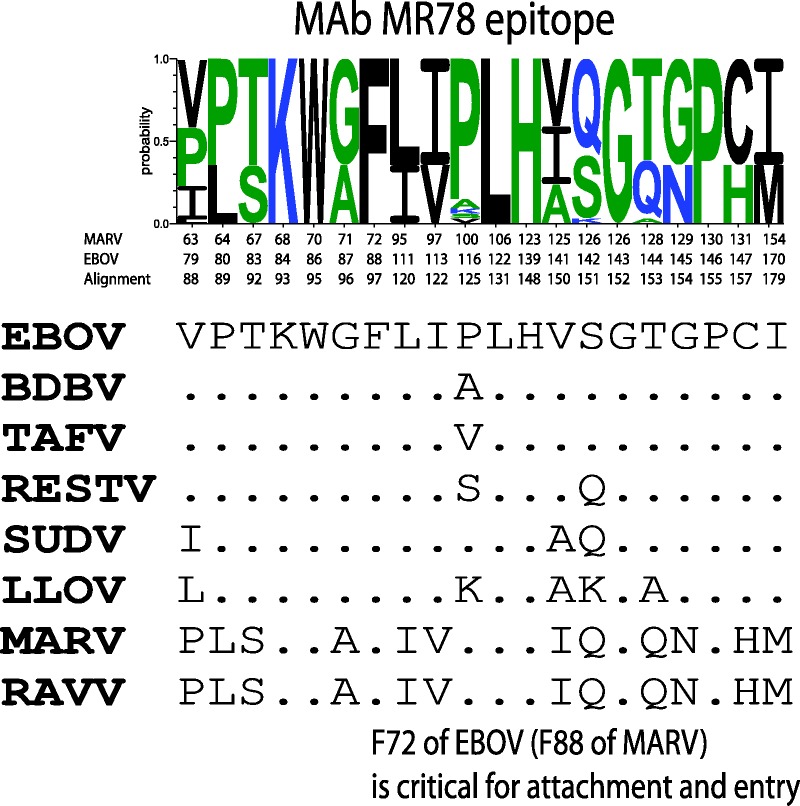
Virus variability across filovirus species of MAb MR78 epitope. Discontinuous positions of MAb MR78 epitope are shown. A Sequence WebLogo was constructed over 34 sequences from the one-per-outbreak alignment (using the QuickAlign HFV database tool (http://hfv.lanl.gov/content/sequence/QUICK_ALIGNv2/QuickAlign.html), but, since the epitope was completely conserved within each species, only species reference sequences are shown under the logo. Position numbering is given according to EBOV, MARV and the alignment.

Applying the HFV database tools Highlighter and SNAP (47) to our codon aligned outbreak-sequence alignment of HFV protein-coding regions reveals synonymous and non-synonymous substitution patterns across viruses from different species and genera. The top panel of Figure 3 (created with the Highlighter tool) shows silent and non-silent substitutions in the GP gene with respect to the EBOV consensus sequence; the middle panel (created with the SNAP tool) shows cumulative silent and non-silent changes along the GP, codon-by-codon; the bottom panel shows the normalized difference between non-synonymous and synonymous changes (dN-dS), analysed by methods for detecting amino acid sites under selection via the external server Datamonkey (48–50). The projecting highly glycosylated mucin-like domain, which dominates GP surfaces available for host interaction (51), is highly enriched for non-synonymous substitutions; this is consistent with positive selection, possibly resulting from ongoing immune pressure. Despite high filovirus GP variability, up to 70% in protein sequence (52, 53), several regions stand out by all three methods as negatively selected throughout all species and genera, suggesting strong functional constraints (Figure 3). These regions overlap several cross-reactive CTL and B cell epitopes, most notably the conformational epitope of cross-reactive neutralizing antibody MR78 from a recent human survivor of MARV infection. This is the first isolated cross-reactive antibody that binds both MARV and EBOV GPs (53, 54), and it overlaps a region of negative selection across filoviruses (Figure 3). The epitope is well conserved across ebolaviruses and there are critical positions conserved across all filoviruses (Figure 4, created with the HFV database tool Quickalign). Similar conserved negatively selected regions are evident in all filovirus genes aligned to EBOV consensus sequence (Supplementary Figure S8A and B).

## Discussion

The HFV database provides carefully annotated and curated nucleotide and protein sequences, and multiple sequence alignments at the *Filoviridae* family, genus, species and outbreak levels. Our Ebola genome browser displays the functional domains and immunological epitopes with detailed information describing how each epitope was first characterized, vaccines used to elicit immune responses, continuous and conformational antibody epitope binding details, notes on structure, cross-reactivity, neutralization, therapeutics and protection. In addition, the database has about 30 computational tools for analysis, many of which are tailored to filoviruses specifically. The unique utility of this database is the level of integration of published sequence, immunological and epidemiological data, and the provision of enabling computational tools for analysis. The integrated data, with metadata concisely incorporated into the sequence names, enables user to rapidly explore hypotheses regarding filovirus sequence variability and geographical and temporal distributions.

The discovery of integrated filovirus elements in mammalian DNA (55, 56) indicates that filoviruses have been co-evolving with mammals for millions of years, an ancient interaction in which integrated filovirus elements have been posited to perhaps confer survival advantage in the host (55, 56). Filovirus genomes sampled in recent decades seem to represent three genera in a very old lineage: estimates of the minimal time to the most recent ancestor of known filoviruses vary widely, from 10 000 (42) to 150 000 years (57), to millions of years (58). As described in Results (Figure 1), phylogenetic estimates of evolutionary rates based on genetic distances between isolates from different human EVD and MVD outbreaks from the last 40 years are complicated by spatial and sampling issues, and it is known that purifying selection and mutational saturation can cause divergence times of recently sampled pathogens to be underestimated (59). Despite these cautionary notes, better understanding of the evolution of these viruses (42) merits continued serious effort. As new sequences accumulate, we hope the field will find the fully annotated alignments provided here a useful baseline to facilitate continued exploration of the evolutionary history and trajectory of these viruses.

From a public health perspective, known HFV protein variation can inform next-generation vaccine design to help protect against new disease outbreaks in an uncertain future, and to help assess the potential breadth of current therapeutics. Ideally, vaccine protection should be sufficiently broad to protect against viruses from both known and unknown species—although most historic outbreaks have been due to EBOV, SUDV or MARV (Figure 1), the BDBV, first discovered in an outbreak in 2007 (a full 30 years after the first known EVD outbreak in 1976), belongs to a distinct species and provides an example why we need to be prepared for the unexpected. T-cell vaccine responses offer a very promising vaccine strategy for Ebola (60, 61), and an attractive feature of T cell vaccine responses over antibodies is they may be elicited against the most conserved domains in relative conserved filovirus proteins (Figure 3). T-cell responses to such highly conserved regions may not merely target known species, but have cross-reactive potential against unknown filoviruses that are subject to the same biological constraints (52). Conserved region T-cell vaccine constructs are immunogenic and can target infected cells in the context of HIV-1, a highly variable virus for which such an approach is being considered for a universal vaccine component (62– 65). Vaccines to elicit antibody responses by necessity will need to target the highly variable GP protein (Figure 3), where cross-species breadth becomes more challenging, but not insurmountable (53, 66– 68). In summary, our updated database provides a useful resource to facilitate and accelerate vaccine and therapeutic research, as well as to provide richly annotated baseline alignments and coordination with reference sequence numbering.

## Supplementary Material

Supplementary Data
