# Supplementary material for: Integrated sequence and immunology filovirus database at Los Alamos
Source: Database (Oxford). 2016 Apr 21;2016:baw047. doi: 10.1093/database/baw047 (PMC4839628; doi:10.1093/database/baw047)
Supplement: Supplementary Data [file supp_2016_baw047_index.html]

Supplementary Data 

# Integrated sequence and immunology filovirus database at Los Alamos

## Supplementary Data

files

- Supplementary Data - zip file
