# Supplementary material for: Integrated sequence and immunology filovirus database at Los Alamos
Source: Database (Oxford). 2016 Apr 21;2016:baw047. doi: 10.1093/database/baw047 (PMC4839628; doi:10.1093/database/baw047)
Supplement: Supplementary Data [file supp_baw047_suppl_data.zip › SupplementalDataRevised.docx]

**Supplemental Figures**

**Figure S1**. **World map showing nations impacted by (A) Ebolavirus and (B) Marburgvirus*.*** While known human outbreaks resulting from zoonosis to date have originated in Equatorial and Western Africa, other countries have been affected through indirect routes (infected people traveling, or import of non-human primates). Such incidents have in some cases resulted in active viral transmission within the human population. The maps show overlapping categories of infection, from primary zoonoses (greatest color intensity), to laboratory-accident infections (least color intensity). For countries with multiple categories (e.g., a country with primary zoonosis and human-to-human transmission), the most intense color is used. The marburgvirus map contains an inset (lower right) showing current boundaries for the countries affected by the 1967 simultaneous outbreaks in Marburg (Germany) and Belgrade (Serbia). The approximate position of this insert is shown as a quadrangle on the main map. Reprinted from the Los Alamos HFV database, <http://hfv.lanl.gov/content/sequence/HFV/RESOURCES/outbreak_maps/outbreak_maps.html>. Data were taken from [references](http://hfv.lanl.gov/content/sequence/HFV/RESOURCES/outbreak_maps/outbreak_maps.html#refs) below, as of February 2015.

[CDC Outbreaks Chronology: Ebola Virus Disease](http://www.cdc.gov/vhf/ebola/outbreaks/history/chronology.html)

[CDC Ebola Virus Disease Distribution Map](http://www.cdc.gov/vhf/ebola/outbreaks/history/distribution-map.html)

[CDC Chronology of Marburg Hemorrhagic Fever Outbreaks](http://www.cdc.gov/vhf/marburg/resources/outbreak-table.html)

[CDC Marburg Hemorrhagic Fever Distribution Map](http://www.cdc.gov/vhf/marburg/resources/distribution-map.html)

**Figure S2. Maximum likelihood tree including one representative strain per human filovirus outbreak**. Viruses from two sequenced filovirus species that have not been isolated in human (RESTV and LLOV), are also included and labeled in black. Sequences are named according to the convention in the Los Alamos Filovirus database: The name starts with a two letter code (noted in the key) to indicate the filovirus species, followed by: the two letter country code according to the ISO international standards (<https://www.iso.org/obp/ui/#search>), the date of sampling (2-digit month/day/year, x if unknown), patient survival status (d indicates death, s survival), the species from which the sample was isolated, isolation type (‘wt’ for virus directly sequenced from a clinical specimen, ‘tc’ for virus that had undergone tissue/cell culture passaging, ‘la’ for virus adapted in the laboratory to cell or animals it would not normally infect, ‘xx’ if unknown), geographical regional information regarding the place the sample was collected, sequence name and GenBank accession number. Only coding regions were including in this analysis. Coding regions are readily aligned, although there were still some insertions and deletions evident. Sequences in the alignment were gap-stripped, leaving only homologous regions that were found across all filoviruses, thus this tree is based on 13560 of the approximately 19,000 bases found in a full length filovirus genome. The maximum likelihood (ML) tree shown here was built using the PHYML ([1](#_ENREF_1)) interface at the Ebola HFV database (<http://hfv.lanl.gov/content/sequence/PHYML/interface.html>). Starting with a neighbor joining tree, the maximum likelihood tree was determined using a GTR model that estimated relative rate parameters, nucleotide frequencies, and the gamma shape parameter (0.815) from the data, using 4 categories of rate variation. This ML tree is presented along with the outbreak map in in Fig. 1.


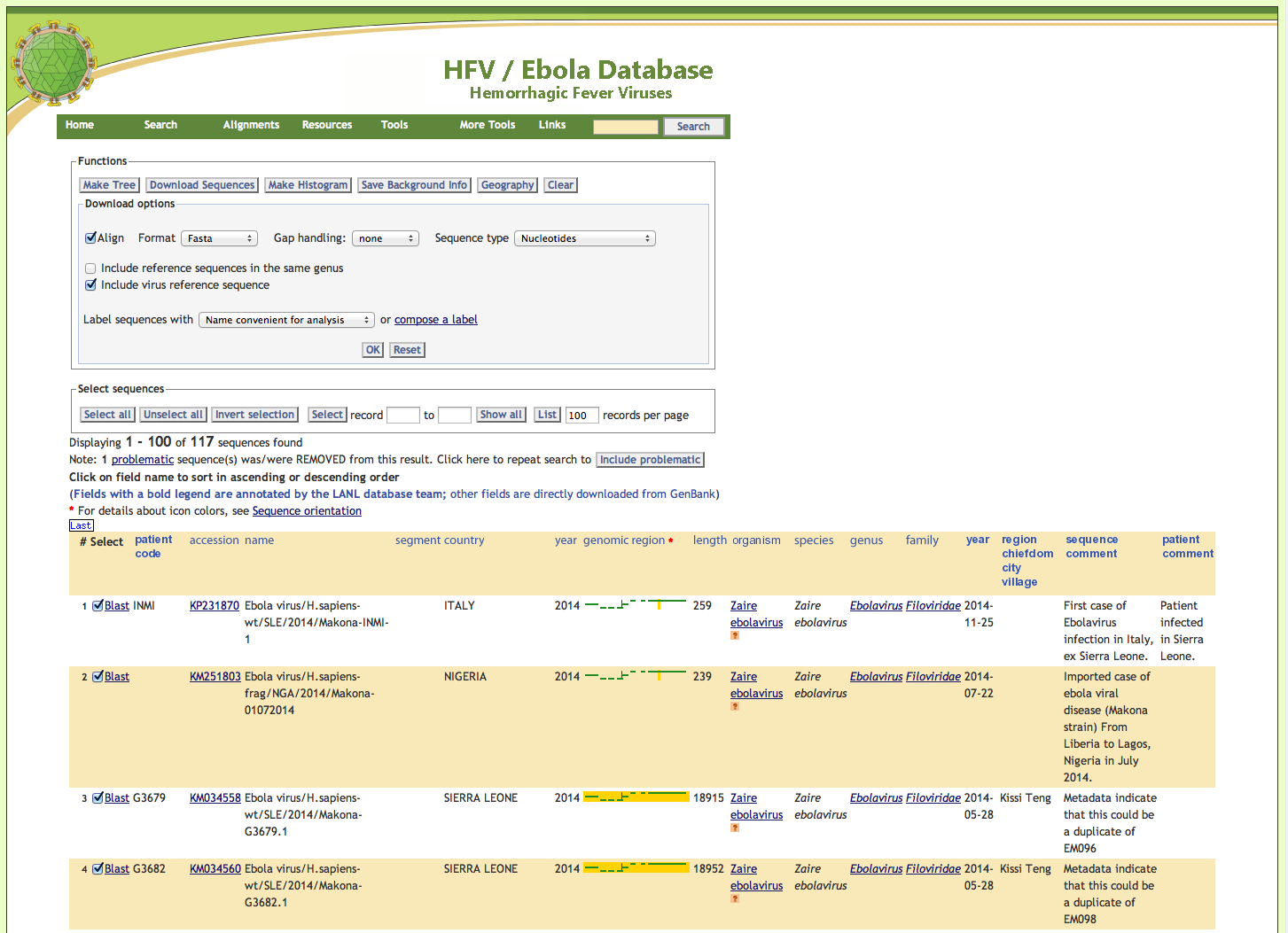


**Figure S3. Snapshot of the sequence search results.** (<http://hfv.lanl.gov/components/sequence/HCV/search/searchi.html>) Multiple fields available for each sequence include BLAST search against HFV database sequences and the fields directly downloaded from GenBank, such as patient code, accession number, sequence name, country, year, genomic region, length, virus name, species, genus, family. Additionally, fields with legends in bold-face type are annotated by the LANL database team from the literature, and include patient outcome, exact date of disease onset, and the most specific geographical location available (e.g., region / chiefdom / city / village), as well as sequence and patient comments. Links are provided for real-time phylogenetic tree reconstruction, the histogram showing the distribution of sequences from the query across the entire genus (Fig. S4), various options for selecting and deselecting the sequences, and options for download. The sequences can be downloaded with names in different formats: preselected fields such as name, virus, seqment, accession; user defined labels where user can decide which fields to include, as well as “modified standardized name” and “name convenient for analysis”. The “modified standardized name” is based on the latest nomenclature ([2-5](#_ENREF_2)) and has the following fields: Virus (4-5 letters)/ Host-isolation (8 letters) / Country (3 letters) / Year (2 letters) / Variant-Isolate / Accession (example: EBOV/H.sap-wt/SLE/14/Makona-G3846/KM233109). The “name convenient for analysis” is based on the standard name, but is further modified for convenience: the virus name has been abbreviated to 2 letters; most of the fields have a consistent number of characters, to aid in parsing; each field has been kept as short as possible; the slash symbol "/" is replaced with the period "." because the slash symbol is problematic for some bioinformatics tools; the period was removed from host species name (i.e., Hsap) to allow for accurate parsing of names into fields; the isolation type has been shortened to 2 characters; the country name has been shortened to the ISO standard 2-letter country code; the date field has been expanded to include the full date, when known; the name includes a field for the patient's survival status, the most distinguishing geographical location is noted in the name (region, chiefdom, city, village). Fig. S2 is an example of using sequences with name convenient for analysis.

**
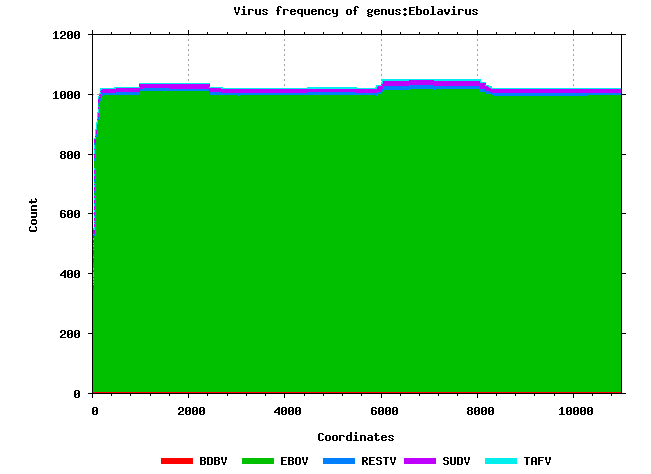
**

**Figure S4. Graphical representation of the number of ebolavirus sequences in the database along the genome.** This histogram shows the distribution of sequences from the query across the entire genus: ebolavirus genome. At each position across the genome, the number of sequences overlapping with that position is plotted. The colors represent different species.

**Figure S5. Geography search pages for filovirus sequences.** A) The global search interface, clicking on a pie chart color, where each species is represented by a color, retrieves the sequences from the geographic region indicated. Clicking on a continent brings up a similar map of the continent, and species are indicated by country; the Africa map is shown in B). <http://hfv.lanl.gov/components/sequence/HFV/geo/make_filo_geo.comp>

**
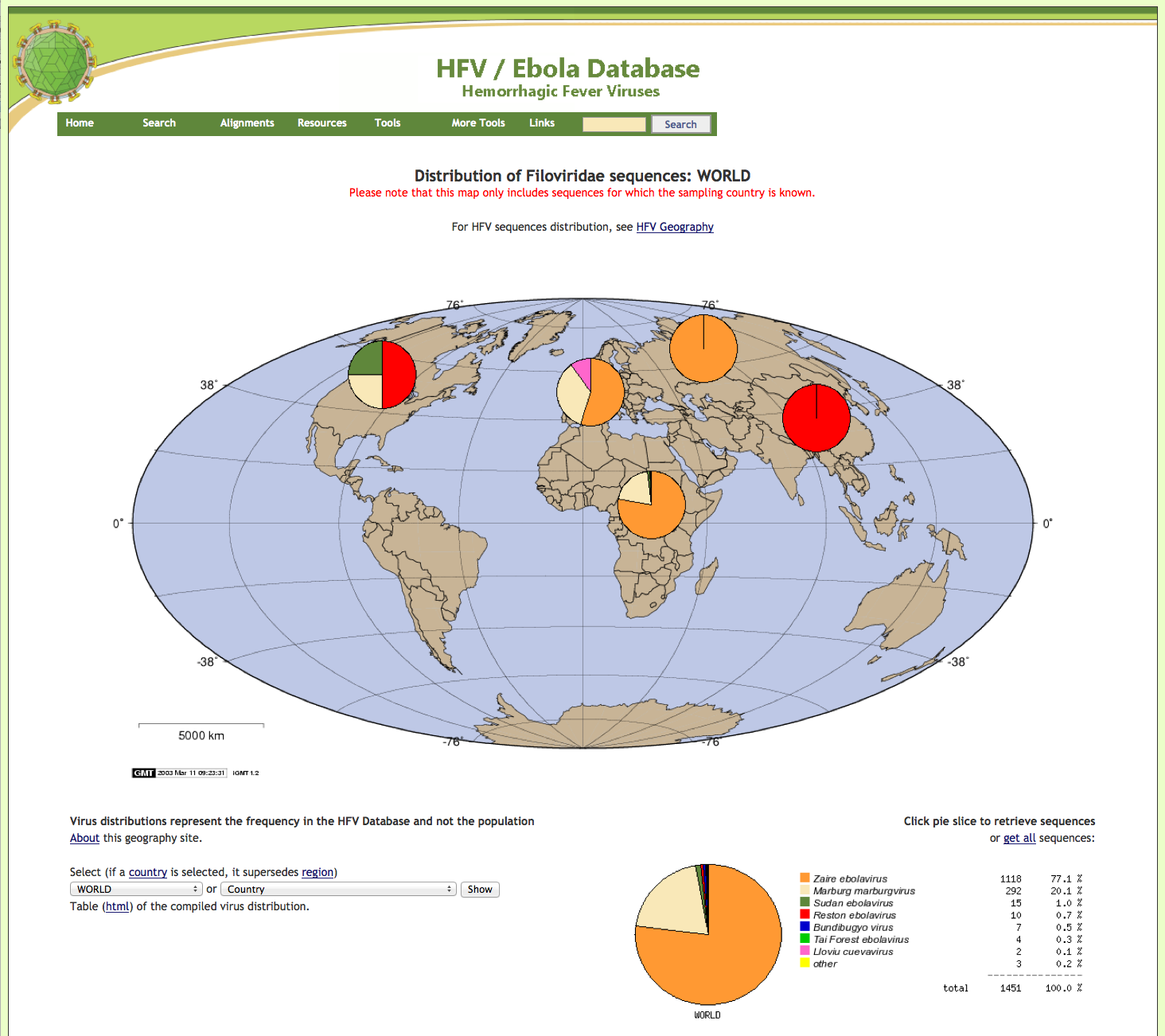
**

**Figure S5A**

**
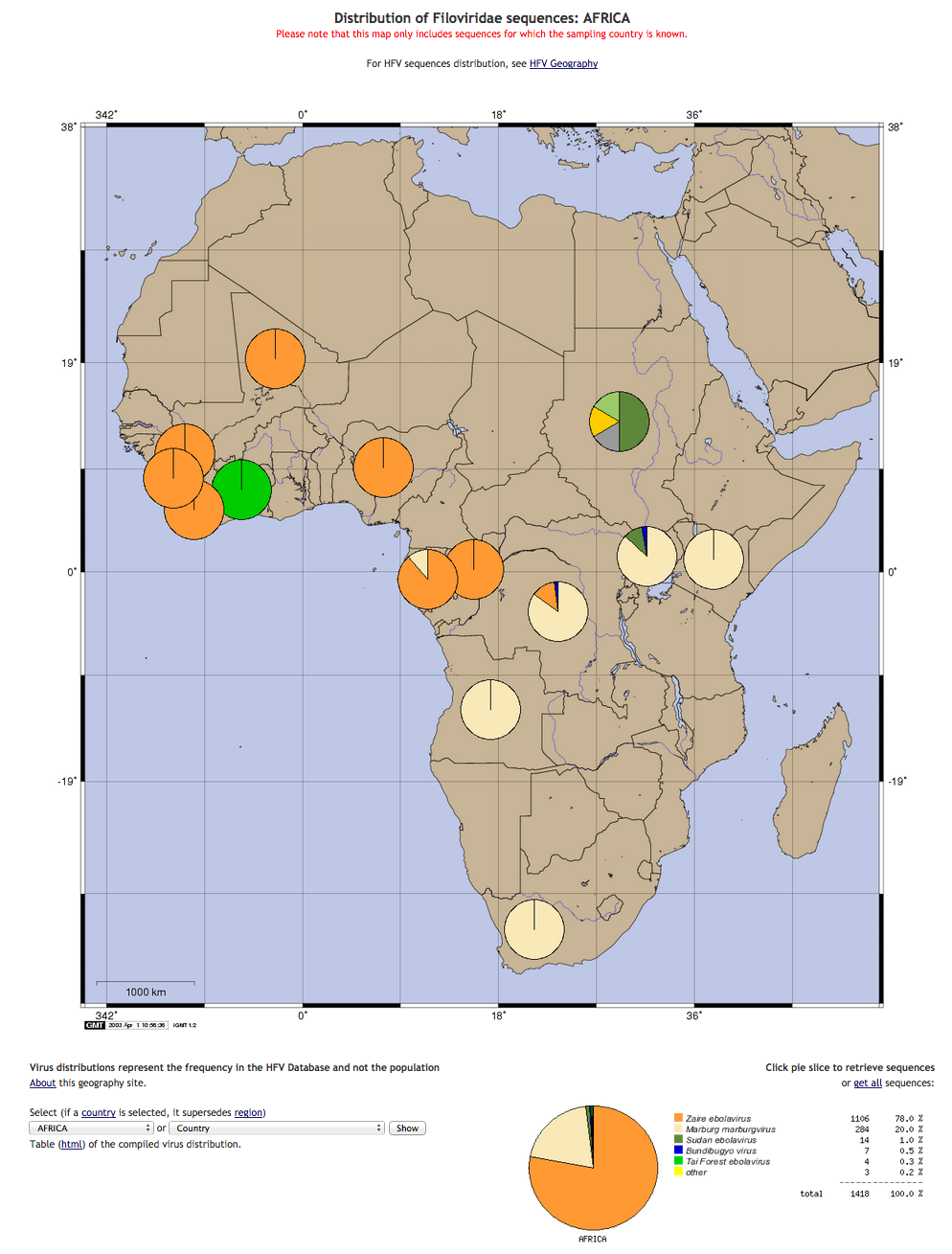
Figure S5B**


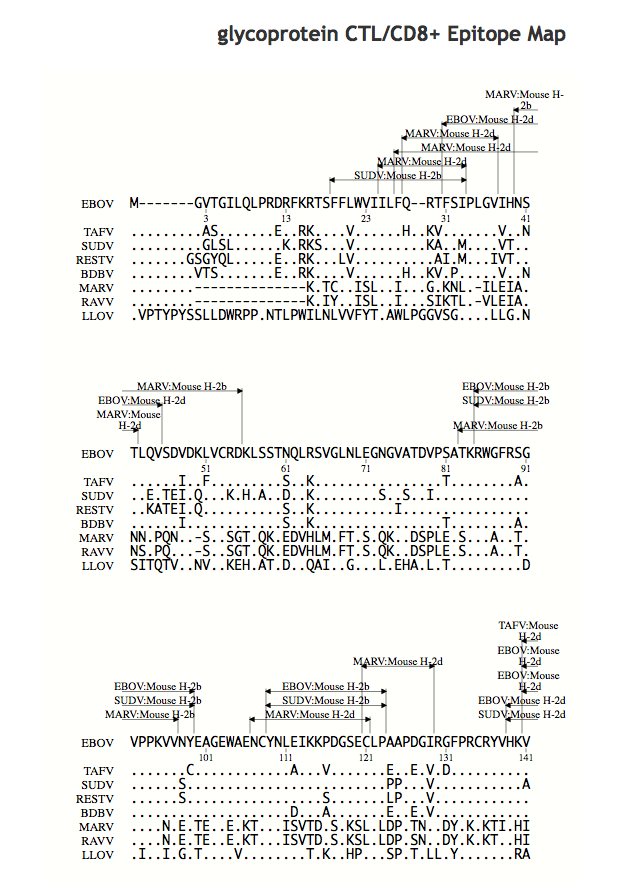


**Fig. S6. Epitope maps.** [**http://hfv.lanl.gov/content/sequence/HFV/RESOURCES/maps/index.html**](http://hfv.lanl.gov/content/sequence/HFV/RESOURCES/maps/index.html)**.** Graphical view of the epitopes and antibody binding sites. Each epitope is presented in its appropriate location relative to the reference sequence, [EBOV Mayinga](http://hfv.lanl.gov/content/sequence/HFV/ebola_names.html). Alignment of reference sequences from each filovirus species is shown, with sequence matches to EBOV shown as dots, and mismatches shown as actual amino acids. Gaps are shown by dashes. Epitope data are also available as spreadsheets (http://hfv.lanl.gov/content/sequence/HFV/RESOURCES/Ebola_immunology_resources.html).

F**igure S7. Phylogeny of just EBOV sequences using the 1976 earliest isolate as outgroup.** The two representative 2014 sequences are shown in bold, one from epidemic in Western Africa, one from the Democratics Republic of Congo.

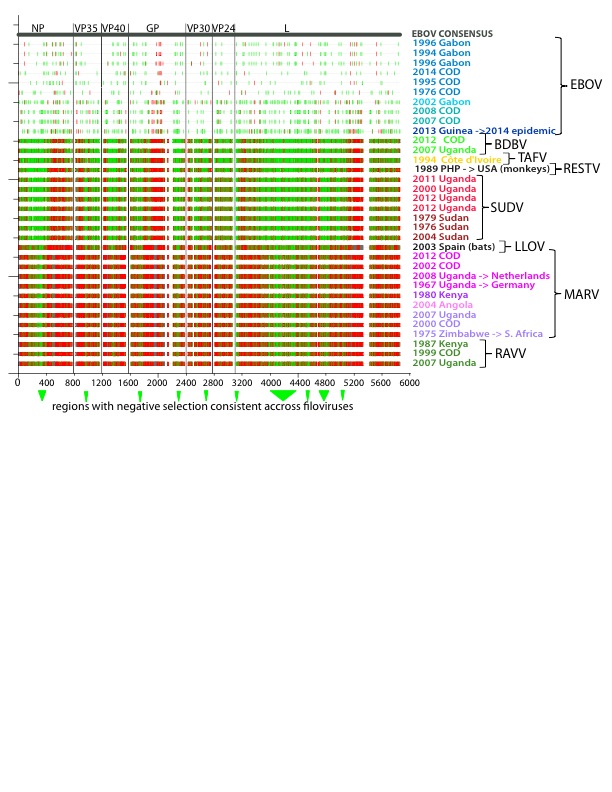


A

B

**Fig. S8. Analysis of synonymous/non-synonymous substitution changes in all filovirus genes. Similar to the top panel of Fig. 3, but plotted for all concatenated filovirus genes.** Uses the same set of 34 one-per-outbreak sequences and is created with the HFV database tool Highlighter (http://hfv.lanl.gov/content/sequence/HIGHLIGHT/highlighter_top.html ). Synonymous (green) and non-synonymous (red) substitutions in each sequence with respect to EBOV consensus sequence are shown by vertical bars. Grey bars represent insertions and deletions. Codon numbering corresponds to the alignment numbering. Green horizontal arrows under the figure represent regions with negative selection (prevalent synonymous substitutions) across filovirises. A) Sequences are compared to EBOV consensus. B) Sequences are compared to MARV consensus. The regions with prevalent synonymous substitutions across filoviruses (negative selection) stay the same irrespective to the choice of the baseline sequence (EBOV or MARV consensus) other sequences are compared to.

**Supplemental data references**

1. Guindon, S., Dufayard, J.F., Lefort, V., Anisimova, M., Hordijk, W. and Gascuel, O. (2010) New algorithms and methods to estimate maximum-likelihood phylogenies: assessing the performance of PhyML 3.0. *Syst Biol*, **59**, 307-321.

2. Kuhn, J.H., Bao, Y., Bavari, S., Becker, S., Bradfute, S., Brauburger, K., Rodney Brister, J., Bukreyev, A.A., Cai, Y., Chandran, K. *et al.* (2014) Virus nomenclature below the species level: a standardized nomenclature for filovirus strains and variants rescued from cDNA. *Arch Virol*, **159**, 1229-1237.

3. Kuhn, J.H., Bao, Y., Bavari, S., Becker, S., Bradfute, S., Brister, J.R., Bukreyev, A.A., Chandran, K., Davey, R.A., Dolnik, O. *et al.* (2013) Virus nomenclature below the species level: a standardized nomenclature for natural variants of viruses assigned to the family *Filoviridae*. *Arch Virol*, **158**, 301-311.

4. Kuhn, J.H., Becker, S., Ebihara, H., Geisbert, T.W., Johnson, K.M., Kawaoka, Y., Lipkin, W.I., Negredo, A.I., Netesov, S.V., Nichol, S.T. *et al.* (2010) Proposal for a revised taxonomy of the family *Filoviridae*: classification, names of taxa and viruses, and virus abbreviations. *Arch Virol*, **155**, 2083-2103.

5. Kuhn, J.H. and Jahrling, P.B. (2010) Clarification and guidance on the proper usage of virus and virus species names. *Arch Virol*, **155**, 445-453.
