# Supplementary material for: Integrated sequence and immunology filovirus database at Los Alamos
Source: Database (Oxford). 2016 Apr 21;2016:baw047. doi: 10.1093/database/baw047 (PMC4839628; doi:10.1093/database/baw047)
Supplement: Supplementary Data [file supp_baw047_suppl_data.zip › Table S1.docx]

**Table S1. Computational tools at Los Alamos HFV Database**

| **Tool** | **Description** | **URL** |
| --- | --- | --- |
|  |  |  |
| **Sequence Searches** |  |  |
| Search Interface, Ebola | Retrieves Filovirus virus sequences | <http://hfv.lanl.gov/components/sequence/HCV/search/searchi_filo.html> |
| Search Interface, HFV | Retrieves sequences for all hemorrhagic fever viruses | <http://hfv.lanl.gov/components/sequence/HCV/search/searchi.html> |
| Advanced Search | Creates a customized search interface | <http://hfv.lanl.gov/components/sequence/HCV/asearch/map_db.comp> |
|  |  |  |
| **Alignments** |  |  |
| Ebolavirus, Marburgvirus and Cuevavirus Premade Alignments | Manually optimized alignments for the Filoviridae | <http://hfv.lanl.gov/content/sequence/NEWALIGN/align.html> |
| HFV Premade Alignments | Manually optimized genus reference alignments for all hemorrhagic fever viruses | http://hfv.lanl.gov/content/sequence/NEWALIGN/align_hfv.html |
|  |  |  |
| **Immunology Resources** |  |  |
| Ebola Immunology Resources | Information about Ebola T-cell epitopes and antibodies | <http://hfv.lanl.gov/content/sequence/HFV/RESOURCES/Ebola_immunology_resources.html> |
| Epitope Maps | Show the location of Ebolavirus and Marburgvirus epitopes, relative to reference sequences | <http://hfv.lanl.gov/content/sequence/HFV/RESOURCES/maps/index.html> |
|  |  |  |
| **Geography Resources** |  |  |
| Global Outbreak Maps | Global distribution of Ebolavirus & Marburgvirus diseases | <http://hfv.lanl.gov/content/sequence/HFV/RESOURCES/outbreak_maps/outbreak_maps.html> |
| Geography Search, Ebola | Retrieves Filovirus sequences based on geographical distribution | <http://hfv.lanl.gov/components/sequence/HFV/geo/make_filo_geo.comp> |
| Geography Search, HFV | Retrieves HFV sequences based on geographical distribution | <http://hfv.lanl.gov/components/sequence/HCV/geo/geo.comp> |
|  |  |  |
| **Genome Coordinate Resources** |  |  |
| Ebola Annotation Spreadsheets | Genome coordinates for features of Filoviridae sequences and various spreadsheet data | <http://hfv.lanl.gov/content/sequence/HFV/RESOURCES/Ebola_data.html> |
| Ebola Coordinates and Naming | Information about the ebola reference sequence and the sequence naming systems used by this website | <http://hfv.lanl.gov/content/sequence/HFV/ebola_names.html> |
|  |  |  |
| **Sequence Manipulation Tools** |  |  |
| AnalyzeAlign | Shows weblogos, calculates frequency by position, and finds variants in an alignment. | <http://hfv.lanl.gov/content/sequence/ANALYZEALIGN/analyze_align.html> |
| CodonAlign | Takes nucleotide alignment and returns codon alignment and translates to protein sequences. | <http://hfv.lanl.gov/content/sequence/CodonAlign/codonalign.html> |
| Consensus | Computes a customizable consensus | <http://hfv.lanl.gov/content/sequence/CONSENSUS/consensus.html> |
| Gapstreeze | Removes columns with more than a given % of gaps | <http://hfv.lanl.gov/content/sequence/GAPSTREEZE/gap.html> |
| ElimDupes | Removes duplicate sequences from an alignment | <http://hfv.lanl.gov/content/sequence/ELIMDUPES/elimdupes.html> |
| HFValign | Aligns your sequence(s) using either the multiple alignment program MAFFT, or using the appropriate hidden Markov model. | <http://hfv.lanl.gov/content/sequence/VIRALIGN/viralign.html> |
| PeptGen | Generates overlapping peptides from a protein sequence | <http://hfv.lanl.gov/content/sequence/PEPTGEN/peptgen.html> |
| Pixel | Generates a PNG image of an alignment using 1 or more colored pixel(s) for each residue | <http://hfv.lanl.gov/content/sequence/pixel/pixel.html> |
| QuickAlign | Aligns short nucleotide or protein sequences (e.g., primers, epitopes) to our prebuilt genome or protein alignments, or to a user alignment | <http://hfv.lanl.gov/content/sequence/QUICK_ALIGNv2/QuickAlign.html> |
| Sequence Locator | Aligns your nucleotide or protein sequence to a reference genome and shows feature and coordinate information | <http://hfv.lanl.gov/content/sequence/HFV/LOCATE/locate.html> |
|  |  |  |
| **Format and Display Tools** |  |  |
| Ebola Genome Browser | Provides interactive views of the Ebola genome and proteome | <http://hfv.lanl.gov/content/sequence/genome_browser/browser_ebola.html> |
| Genome Mapper | Creates a genomic feature map for any HFV organism | <http://hfv.lanl.gov/content/sequence/HFV/GenomeMapper/GenomeMapper.html> |
| Format Converter | Converts between 18 standard sequence formats | <http://hfv.lanl.gov/content/sequence/FORMAT_CONVERSION/form.html> |
| SeqPublish | Makes publication-ready alignments | <http://hfv.lanl.gov/content/sequence/SeqPublish/seqpublish.html> |
| Protein Feature Accent | Provides an interactive 3-D graphic of selected HFV proteins; the user can map a sequence feature and see where it occurs spatially. | <http://hfv.lanl.gov/content/sequence/PROTVIS/html/protvis.html> |
| Analysis Tools |  |  |
| HFV BLAST | Finds sequences similar to yours in the HFV database. | <http://hfv.lanl.gov/content/sequence/BASIC_BLAST/basic_blast.html> |
| Highlighter | Shows matches, mismatches and mutations in an aligned set of nucleotide sequences. | <http://hfv.lanl.gov/content/sequence/HIGHLIGHT/highlighter_top.html> |
| N-Glycosite | Finds potential N-linked glycosylation sites in a protein alignment | <http://hfv.lanl.gov/content/sequence/GLYCOSITE/glycosite.html> |
| PCOORD | Provides multidimensional analysis of sequence variation | <http://hfv.lanl.gov/content/sequence/PCOORD/PCOORD.html> |
| SNAP | Calculates synonymous/non-synonymous substitution rates | <http://hfv.lanl.gov/content/sequence/SNAP/SNAP.html> |
| VESPA | Detects signatures: residues with different frequencies in two sequence sets | <http://hfv.lanl.gov/content/sequence/VESPA/vespa.html> |
|  |  |  |
| **Phylogenetics Tools** |  |  |
| FindModel | Finds which evolutionary model best fits your sequences | <http://hfv.lanl.gov/content/sequence/findmodel/findmodel.html> |
| PhyloPlace | Reports phylogenetic relatedness of query sequence with reference sequences in known clades. | <http://hfv.lanl.gov/content/sequence/phyloplace/PhyloPlace.html> |
| PhyML interface | Creates and displays a Maximum-Likelihood tree using the PhyML code; many evolutionary models can be specified | <http://hfv.lanl.gov/content/sequence/PHYML/interface.html> |
| Rainbow Tree | Color codes phylogenetic tree branches according to labels in the sequence names | <http://hfv.lanl.gov/content/sequence/RAINBOWTREE/rainbowtree.html> |
| TreeMaker | Generates a quick-and-dirty phylogenetic tree | <http://hfv.lanl.gov/content/sequence/TREEMAKER/TreeMaker.html> |
| TreeRate | Calculates branch lengths between internal and end nodes based on a Newick treefile | <http://hfv.lanl.gov/content/sequence/TREERATEv2/treerate.html> |
|  |  |  |
